# Association between self-reported hearing impairment and diabetes: a Brazilian population-based study: Association between self-reported hearing impairment and diabetes in adults

**DOI:** 10.1186/s13690-018-0300-6

**Published:** 2018-09-20

**Authors:** MO Soares, NSX Oenning, PK Ziegelmann, B N G Goulart

**Affiliations:** Graduate Program in Epidemiology, Universidade Federal do Rio Grande do Sul, Rua Ramiro Barcelos, 2400, 2° andar, Porto Alegre, Rio Grande do Sul Brazil

**Keywords:** Diabetes mellitus, Hearing loss, Survey, Prevalence, Epidemiology, Occupational, Associated factors

## Abstract

**Background:**

Some studies have already explored the relationship between diabetes and hearing loss; however, this relationship has still not been well established, especially due to methodological limitations related to lack of control for confounders. The aim of this study was to analyze the association between self-reported hearing impairment and diabetes among adults in Brazil, controlling for sociodemographic and occupational exposure to ototoxic agents.

**Methods:**

This is a cross-sectional study based on data collected by the National Health Survey of 2013 in Brazil. A total of 60,202 individuals aged≥18 years were interviewed. Crude and adjusted prevalence ratios were calculated using the Poisson regression model with robust estimation of the variance. All analyzes were performed considering the appropriated weights imposed by the complex sample design.

**Results:**

Hearing loss prevalence was 2.56% (95%CI: 2.34–2.79). It was higher in males, older age groups, white and individuals with lower levels of schooling. Diabetes was positively and significantly associated with hearing loss in the crude analysis (PR_crude_ = 2.92; 95%CI: 2.75–3.11) and also in the analysis adjusted for gender, age, skin color, schooling, smoking, alcohol consumption and occupational exposure (PR_adj_ = 1.46; 95%CI: 1.32–1.61).

**Conclusions:**

The present results suggest that individuals with diabetes have higher prevalence of hearing impairment. There is the need of longitudinal studies to investigate if diabetes is a risk factor to hearing impairment.

## Background

Estimates indicate that more than 5% of the population, approximately 360 million people in the world, have hearing impairment in some extent [1]. Disabling deafness refers to hearing loss greater than 40 decibels (dB) in the best ear in adults, and hearing loss greater than 30 dB in the best ear in children. Most people with incapacitating hearing impairment live in low and middle-income countries, and approximately one third of people over 65 years of age are affected by incapacitating deafness [1].

On the other hand, diabetes has been a major cause of morbidity and mortality and one of the four major chronic diseases identified by the World Health Organization (WHO) as a focus for control and prevention [2]. It was responsible for 4.9 million deaths worldwide in 2014 and for 11% of the total adult health expenditure, estimated at $ 612 million [3].

Audiometry is the gold standard for the detection of hearing loss but performing it in population surveys becomes complex and costly. This procedure requires booth, audiometric equipment and trained personnel. Thus, self-referred population surveys that verify the prevalence of hearing impairment through a simplified questionnaire may be an alternative and have been ratified in studies that found acceptable sensitivity and specificity values when compared to the gold standard [4].

Studies with self-declared surveys about the hearing condition can provide clues about temporal trends in the prevalence of hearing loss, contributing to the identification of groups at greater risk. They are also characterized as faster and more economical surveys to provide population estimates, since they minimize fieldwork time and costs with equipment and trained professionals [5].

These two public health problems, namely hearing impairment and diabetes, seem to be associated, especially over the fourth decade of life. Authors justify that hearing loss and its associated factors, such as tinnitus and vertigo, may be associated with diabetes due to microcirculatory insufficiency regarding a vascular occlusion by embolism, hemorrhage or vasospasm which, in turn, would be due to a hyperviscosity syndrome or microangiopathy (due to diabetes or even due to hypertension) [6, 7].

The global burden of diabetes is expected to increase due to higher longevity and life style changes in the population. Those characteristics will adversely affect the patient’s quality of life and their ability to live independently [8]. Also, considering that hearing impairment is associated with diabetes and that this is a major public health problem in low and middle-income countries and in developed economies, it is important to develop population-based studies in order to know the magnitude of this association.

Surveys already conducted [9–13] have demonstrated diabetes as one of the predisposing factors for hearing loss, but it was found methodological fragility regarding these studies. Variables that may be related to hearing loss, such as aspects related to lifestyle and occupation, which may be potential triggers of hearing impairment, were not considered in the studies. In our research, we adjusted the results for those variables. And to our knowledge, this is one of the first national studies with a population basis that intends to analyze this issue.

The National Health Survey (*Pesquisa Nacional de Saúde* - PNS) is a survey with national representativeness from which it is expected consistent data in order to contribute to the elaboration, evaluation and monitoring of actions and health programs for the Brazilian population. It is expected that hearing care and attention to diabetes will gain greater attention after the results provided in this study.

This study aimed to analyze the association between self-reported hearing impairment (SHI) and diabetes among adults in Brazil, based on self-reported data from the National Health Survey (PNS) of 2013 considering adjustments factors (gender, age, skin color, schooling, smoking, alcohol consumption and occupational exposure).

## Methods

### Study design

This is a cross-sectional study with data from the PNS, a household survey carried out in Brazil in 2013 by the Brazilian Institute of Geography and Statistics (IBGE) in partnership with the Ministry of Health (MS) and the Instituto Oswaldo Cruz Foundation (Fiocruz).

### Population and sample

The targed population was adults aged 18 years or over living in private households throughout the Brazilian territory, excluding the special census tracts (barracks, military bases, lodges, camps, boats, penitentiaries, penal colonies, prisons, jails, asylums, orphanages, convents and hospitals). Cluster sampling was used, divided into three stages, with the census tracts as primary units, households as secondary units, and a tertiary unit composited by an adult (≥18 years old) selected from each household to answer the applied questionnaire. The detailed methodology is described in a previously published article [14, 15].

A total of 81,167 households were visited, of which 69,994 were occupied. There were 64,348 home interviews and 60,202 individual interviews with the selected resident in the household, which resulted in a non-response rate of 8.1% [14, 15].

### Data collection and questionnaire

Data collection was performed by the IBGE team, that is, interviewers with the support of supervisors and coordinators. The training program and material of the field staff was done in partnership with the MS. The training of coordinators and supervisors for the data collection was on-site and the interviewers were trained by the coordinators and supervisors. Interviews were conducted with the help of handheld computers - PDA *(personal digital assistance),* between August 2013 and February 2014. The interviewers were adequately trained to address the interviews and include the responses in the PDA [14, 15].

The PNS questionnaire is divided into two parts: the first one (home interview) consists of 11 modules and is answered by a representative of the domicile who responds by all residents of the household. The second part (individual interview) is composed of 9 modules and is answered by the resident selected randomly among all eligible adult residents of the household, and consists of questions only for this resident.

### Variables

SHI was the outcome (*“Does_______ have hearing loss?”)* defined as binary (“yes” or “no”) and was answered in the household interviews. All individuals that had responded as being hearing impaired were included in the SHI population. The main exposure considered is the self-reported diagnosis of diabetes: *“Has a doctor ever given you the diagnosis of diabetes?”, defined* as binary (women who responded only during pregnancy were considered as *“no”* for diabetes) and it was not answered by those who said they had never made blood test to measure blood glucose*.* The adjustment factors considered were: sex (female and male), age group (18 to 29 years, 30 to 59 years, 60 to 64 years, 65 to 74 years or ≥ 75 years), skin color (white, black or others), schooling (incomplete elementary school, incomplete elementary school and incomplete middle school, incomplete high school or incomplete higher education), smoking (“yes” or “no”), alcohol consumption (defined in two categories: “yes” or “no”) and occupational exposure (yes, no or no answer).

The occupational exposure adjustment factor was constructed based on a recent systematic review [16]. Firstly, we considered the binary variables: handling chemical substances, exposure to noise (intense noise), and handling with radioactive material (transportation, receiving, storage, working with x-ray), handling with municipal waste (litter) and exposure to industrial marble (dust marble). Association between each of these variables with hearing loss were evaluated. Only the variables handling with chemicals, exposure to noise and handling with urban waste were significantly associated with hearing impairment. Thus, the occupational exposure factor was defined as: yes (individuals who answered “yes” to at least one of the three questions), no (individuals who answered “no” to all of the three questions) or no response (individuals who did not have to answer these questions considering they were classified as “employed”). The “no response” categorie was considered in order not to lose statistical power.

The SHI population studied was characterized by the following variables: type of disability, involvement of the ears, degree of limitation that the disability brings, use of rehabilitation service, use of hearing aid and use of rehabilitation service by individuals using prosthesis. All the variables included in this study were obtained in a self-reported form answered by the respondents.

### Data analysis

The characterization of the sample was presented through absolute and relative frequencies. These frequencies are the result from the extrapolation of the observed data to the true values considering the complex sample weigths. Differences in the distribution of the variableswere performed using the Rao-Scott chi-square test. Prevalence ratio estimates (crude and adjusted) were calculated using the Poisson regression model with robust estimation of the variance. All analyzes were performed considering the effect of the study design. Analyzes were performed using the survey and sandwich packages and the R Version 3.4.1.

## Results

Table 1 describes socio-demographic data, life habits and occupational exposure in Brazilian adults with and without SHI. Among the individuals that reported SHI, the highest percentages were found among women, older elderly, whites, with low level education and individuals who were exposed to occupational risk factors.

**Table 1:** Socio-demographic characeristics, smoking, alcohol and occupational exposure – National Health Survey 2013, Brazil

	Self-reported Hearing Impairment (SHI)						
	**Yes** (*N* = 1464)			**No** (*N* = 58,738)			*p*-value
	n	%	%w	n	%	%w	
Sex							< 0.001
Female	722	2.11	2.23	33,560	97.89	97.67	
Male	742	2.86	2.94	25,178	97.14	97.06	
Age Group (years)							0.000
18–29	76	0.53	0.54	14,245	99.47	99.46	
30–59	551	1.59	1.74	34,153	98.41	98.26	
60–64	134	3.87	4.48	3331	96.13	95.51	
65–74	312	6.47	7.07	4513	93.53	92.93	
≥ 75	391	13.5	14.2	2496	86.46	85.84	
Color or Race							0.002
White	701	2.91	2.9	23,405	97.09	97.1	
Black	122	2.17	1.66	5509	97.83	98.34	
Others	641	2.1	2.38	29,824	97.89	97.62	
Education Level^a^							0.000
Graduated or higher	121	1.41	1.38	8475	98.59	98.62	
Second grade or incomplete undergraduation	242	1.3	1.39	18,347	98.7	98.61	
First grade or incomplete second grade	309	2.02	2.14	14,979	97.98	97.86	
Incomplete first grade	467	6.12	6.32	7163	93.88	93.68	
Smoking Consumption							0.764
No	1226	2.38	2.55	50,247	97.62	97.45	
Yes	238	2.73	2.63	8491	97.27	97.36	
Alcohol Consumption							0.002
No	1030	2.77	2.87	36,170	97.23	97.13	
Yes	434	1.87	2.11	22,568	98.11	97.89	
Occupational Exposure							0.000
No	188	1.08	1.12	17,145	98.91	98.88	
Yes	357	1.87	1.88	18,752	98.13	98.11	
No response	919	3.87	4.22	22,841	96.13	95.78	

%: raw frequency

%w: weighted frequency

^a^ missings (*n* = 50,103)

Table 2 describes the SHI population. There was a higher prevalence of acquired hearing impairment (HI) (91.3%), with reduced hearing (35.6% in both ears and 30.3% in only one year), participants stating that the HI does not limit (37.0%) or little limits (31.2%) them, not using a rehabilitation service (93.4%), not using a hearing aid (87.0%) and not using a reabilitation service in the case of individuals using prosthesis (83.6%).

**Table 2:** Self-reported hearing impairment (SHI) in 1464 adults (≥ 18 years) - National Health Survey 2013, Brazil

Variables	N	Prevalence (%)	95% CI
Type of disability			
Born with disability	139	8.7	6.8–11.1
Acquired	1325	91.3	88.9–93.2
Ear affection			
Deafness in both ears	123	8.2	6.6–10.2
Deafness of one ear and reduced hearing in the other	126	9.6	7.5–12.3
Deafness of one ear and normal hearing in the other	216	16.2	13.8–19.0
Hearing reduced in both ears	547	35.6	32.1–39.2
Hearing reduced in one ear	452	30.3	27.0–33.8
Degree of limitation that disability brings			
Does not limit	537	37.0	33.5–40.7
Little	457	31.2	27.7–35.0
Moderately	287	18.9	16.4–24.7
Intensely	133	10.2	7.9–13.0
Very intensely	50	2.7	1.7–4.2
Use of rehabilitation service			
Yes	77	6.6	4.8–8.8
No	1387	93.4	91.2–95.2
Make use of hearing aid			
Yes	195	13.0	10.6–15.9
No	1269	87.0	84.1–89.4
Use of auditory rehabilitation service in hearing aid users			
Yes	32	16.4	12.8–20.9
No	163	83.6	79.1–87.2

Table 3 shows that the SHI estimated prevalence was 2.56% (95%CI: 2.34–2.79). It also shows that being a male (PR = 1.32; 95%CI: 1.26–1.37), age (prevalence of SHI increases with age), white color or race (PR_black_ = 0.57; 95%CI:0.54–0.60 and PR_others_ = 0.82; 95%CI:0.77–0.87), level of education (prevalence of SHI increases with decreasing schooling), non-consumption of alcohol (PR = 1.35; 95%CI:1.28–1.43) and occupational exposure (PR = 1.68; 95%CI:1.55–1.83) are factors positively associated with SHI. The smoking factor was not significantly associated (PR = 1.03; 95%CI: 0.98–1.08) with SHI.

**Table 3:** Factors associated with self-reported hearing impairment (SHI) - National Health Survey 2013, Brazil

Variables	Prevalence (95%CI)	PR_Crude_ (95% CI)	*P* value
Total	2.56 (2.34–2.79)	–	–
Sex			
Female	2.23 (1.96–2.51)	1.00	
Male	2.94 (2.59–3.28)	1.32 (1.26–1.37)	< 0.001
Age Group			
18–29	0.54 (0.34–0.74)	1.00	–
30–59	1.74 (1.50–1.98)	3.23 (2.91–3.58)	< 0.001
60–64	4.49 (3.18–5.79)	8.33 (7.28–9.53)	< 0.001
65–74	7.07 (5.74–8.40)	13.13 (11.73–14.70)	< 0.001
≥ 75	14.16 (12.02–16.29)	26.30 (23.63–29.26)	< 0.001
Color or Race			
White	2.90 (2.57–3.24)	1.00	–
Black	1.66 (1.19–2.12)	0.57 (0.54–0.60)	< 0.001
Others	2.38 (2.02–2.74)	0.82 (0.77–0.87)	< 0.001
Education Level^a^			
Graduated or higher	1.38 (0.96–1.80)	1.00	
Second grade or incomplete undergraduation	1.39 (1.10–1.67)	1.01 (0.90–1.13)	0.868
First grade or incomplete second grade	2.14 (1.75–2.54)	1.56 (1.39–1.74)	< 0.001
Incomplete first grade	6.32 (5.42–7.23)	4.59 (4.14–5.10)	< 0.001
Smoking Consumption			
No	2.55 (2.31–2.79)	1.00	–
Yes	2.63 (2.11–3.16)	1.03 (0.98–1.08)	0.176
Alcohol Consumption			
No	2.87 (2.56–3.18)	1.00	–
Yes	2.11 (1.79–2.44)	0.74 (0.70–0.78)	< 0.001
Occupational Exposure			
No	1.12 (0.85–1.39)	1.00	
Yes	1.89 (1.53–2.24)	1.68 (1.55–1.83)	< 0.001
No response	4.22 (3.78–4.66)	3.77 (3.51–4.04)	< 0.001

^a^missing (n = 50,103)

The prevalence of diabetes in the SHI population was 6.69% (95%CI: 5.24–8.14) with PR_crude_ = 2.92 (95%CI: 2.75–3.11). The association between diabetes and SHI was adjusted for sex, age, race or skin color, educational level, smoking, alcohol consumption and occupational exposure remained significant (PR_adjusted_ = 1:46, 95%CI: 1.32–1.61) (Table 4).

**Table 4:** Association between hearing loss and diabetes- National Health Survey 2013, Brazil

Diabetes	Prevalence (95%CI)	PR_Crude_ (95% CI)	*P* value	PR_Adjusted_ (95% CI) ^a^	*P* value^a^
No	2.29 (2.07–2.50)	1.00		–	
Yes	6.69 (5.24–8.14)	2.92 (2.75–3.11)	< 0.001	1.46 (1.32–1.61)	< 0.001

^a^Adjusted for sex, age, skin color or race, education level, tobacco consumption, alcohol consumption and occupational exposure risk for hearing loss. PR = prevalence ratio

## Discussion

This study presentes the SHI prevalence and its association with diabetes. The hearing impairment prevalence was higher in men (2.94; 95%CI: 2.59–3.28), from the sixth decade of life (4.49; 95%CI: 3.18–5.79), among those who declared themselves as white skin or color (2.90; 95%CI: 2.57–3.24) and among adults with less schooling (6.32; 95%CI:5.42–7.23).

Population studies on the prevalence of hearing impairment are not numerous, especially when dealing with the Brazilian population and, when available, present different methodologies, an aspect that makes comparison between the findings difficult.

The estimated SHI prevalence was 2.56% (95% CI:2.34–2.79). This value is less than some national studies, as IBGE Census [17], in which the prevalence of hearing loss in the general population was 5.1%.This difference in prevalence can be explained by the fact that the Census evaluation takes into account the entire Brazilian population, including children and adolescents, a population that is not evaluated by PNS, which only counts individuals aged 18 years and over. Or, also, by the fact that the Census considered the presence of the disability while the PNS evaluated it through the question: “Do you have a hearing impairment?”, The Census questioned: “Do you have a permanent hearing problem?”

The National Health Interview Survey (NHIS), 2010 [18], research with methodology similar to the PNS, estimates in 16% the prevalence of hearing impairment among people aged 18 years and over, and in the NHIS 2012 [19], the prevalence decreased to 15%. Even so, it is almost 6 times higher than in Brazil. The NHIS considers four categories: mild hearing loss, moderate hearing loss, severe hearing loss and deafness, whereas this study used a binay variable: yes or no to hearing impairment. Therefore, due to the differences between our study and the former, it is difficult to compare.

In the cross-sectional study by Hong et al. [10] with data from the Korea National Health and Nutrition Examination Survey (KNHANES) 2010–2012, the estimated prevalence of mild hearing loss was 20.5% (95%CI: 19.6–21.6) and moderate to severe was 9.2% (95% CI: 8.6–9.9). The difference between the prevalence of hearing impairment in the Korean study and in the present study was approximately 9 times higher in mild loss and 3 times higher in the case of moderate to severe loss. The main difference in these results may be due to the fact that the Korean study performed the audiometric test in the participants, thus detecting even the smallest hearing loss, which highlights a limiting factor of self-reported studies, as in the present case.

A higher prevalence of hearing impairment among men than among women has been reported in other studies in Brazil [17, 20, 21] and abroad [10]. This finding can be attributed to exposure differences thoughout life; therefore, men tend to play more unhealthy work or are more exposed to noisy environments in their work activities [21]. Intrinsic conditions can also elucidate these differences, since even if the structures of the auditory system seem similar for men and women at birth, small differences were observed in the results of otoacoustic emissions and evoked potentials of the brainstem and, consequently, hormonal and metabolic differences should also be considered in these analyzes [22].

This study showed that, the older the individual, the higher the prevalence of hearing loss. These results were corroborated by other studies conducted in other countries such as the United States [11, 19], Korea [10] and also in Brazil [20, 23]. These findings lead us to consider the growth of the elderly population and consequent increase in the prevalence of hearing loss in Brazil and in the world, as we know that with increasing age auditory acuity decreases [24].

It is estimated that the proportion of individuals aged 60 and over ranged from 5% in 1960 to 8.6% in 2000 and will increase to 14% by 2025, reaching a significant proportion in developed countries [25]. As a result, the prevalence of chronic non-communicable diseases and hearing loss will increase.

Minor differences were observed for skin color or race, with a higher prevalence of hearing loss among adults who declared themselves as white. These results meet the NHIS research in 2012 [19] and NHANES in 1999–2004 [11]. No studies were found that clearly explained the difference between skin color or race and hearing loss, but socioeconomic factors, associated diseases and occupational exposure may be related to it.

In a study that aimed to analyze self-reported differences between blacks and whites [26], regarding limitations in daily life activities, functional limitations, visual and hearing impairment, and memory and learning problems, it was verified that the probability of visual and hearing problems in blacks in older age groups is lower than in whites in both men and women. The study suggests that this difference may be due to racial differences in the incidence of cataracts, the main cause of vision problems among the elderly, where there is a much higher incidence among white people than blacks [27]. As for differences exclusively in hearing, the study did not mention anything.

In the present study, people with less education tend to have a higher prevalence of hearing impairment [11], with higher hearing thresholds, as in the study by Lee et al. [9], in which he mentions that low schooling and low income may be associated with unhealthy lifestyles, which may, in turn, contribute to the risk of hearing loss.

As to the type of hearing impairment, the acquired one was the most prevalent. Across the world, 16% of incapacitating hearing loss in adults is attributed to occupational noise, ranging from 7 to 21% in the various subregions. The effects of noise exposure are higher for men than for women in all sub-regions and higher in developing regions [28]. In a study, Nelson et al. *e*valuated the global burden of hearing loss induced by occupational noise, concluding that occupational noise is an important risk factor for hearing loss in workers at most ages.

Concerning the involvement of the ears, 56.1% of those who self-referred hearing loss have deafness or reduced hearing in one ear. In American study [11] in speech frequencies the prevalence of unilateral hearing loss was 7.9% and bilateral of 7.8%; while at high frequencies that prevalence was 13% unilateral and 19% bilateral.

About the degree of limitation that hearing impairment brings, the present research showed that, for 37.0% (95% CI: 33.5–34.7) of those who have it, it is not a limitation. A percentage of 74.5% reported having no difficulties in leisure activities, 88.6% said they did not need help in daily activities, and 63.3% reported having no need for assistance, although they did not have statistical significance [29].

In this population who reported hearing impairment 87.0% (95%CI: 84.1–89.4) have not made use hearing aids. The hearing rehabilitation service has been used by 16.4% (95%CI: 12.8–20.9) of those who use this device.

In a research of Cruz et al. [30] performed with the elderly, 89.9% of the participants who self-referred hearing loss have not made use of a hearing aid and 10.1% (*n* = 45) have used it. When asked why they have not used the hearing aid, 8.6% said that although indicated, they had not got used to it and 8.0% had not acquired it because of financial problems. Analyzing the ways of acquiring the device, 78.8% had used private resources and 16.9% had acquired it through the Unified Health System. On receiving training for the use of hearing aids, 87% had received it and 81.4% had been followed up for control of the hearing aid.

Smoking not was significant in this study, but one study found nicotinic receptors in the hair cells of the cochlea, suggesting that smoking has detrimental effects on the function of hair cells due to possible action during auditory neurotransmission [31]. In addition, the impact of smoking could interact with other factors or with harmful auditory exposures, such as noise, causing synergistic detrimental effects on auditory function [32, 33].

On the other hand, alcohol consumption was a protective factor for hearing loss in the univariate analysis. In a population-based cross-sectional study of 164,770 adults in the UK aged 40–69 years who completed the “speech-in-noise” hearing test, those who were drinkers were approximately 40% less likely to have a hearing loss than those who do not drink [34].

The study of Upileet al. [35] suggests that the use of alcohol preferentially affects low frequencies, including the 1000 hertz, which is the most important to discriminate vowels. The reduction in hearing these frequencies is more harmful to the understanding of human speech and light to moderate alcohol consumption also affects the auditory thresholds in the frequencies of speech. However, the study of Curhan*et al.* [36], conducted with a cohort of nurses, concluded that alcohol consumption is not associated with the risk of hearing loss in women, corroborating our findings.

As for the occupational exposure variable (chemicals, noise and solid waste management), it proved to be associated with hearing impairment. Noise is the most common occupational exposure addressed in studies and is associated with hearing loss related to work. A survey of US universities gardeners concluded that these professionals have been exposed to excessive noise, exceeding the limits of 85 dB, which can be effectively reduced through careful programming encouraging the use of personal protective equipment [37]. Between Taiwanese workers the prevalence of hearing loss was higher in the group exposed to noise and toluene compared to the reference group [38].

A study with municipal workers of solid waste landfills showed that there were several occupational hazards for these, including exposure to noise, dust, toxic gases, heat, heavy metals and volatile organic compounds [39]. Mohammadi *et al.* [40] found that the combined exposure to organic solvents (including benzene, toluene, xylene, etc.) and noise can exacerbate hearing loss, especially in high frequency (average hearing threshold greater than 25 dB at 3, 4, 6 and 8 kHz).

Another study with municipal workers of solid waste landfills that aimed to investigate occupational hearing loss divided the sample into 3 groups: group 1 of 63 workers without exposure to occupational risks (control group); group 2 of 84 workers with little or short exposure to occupational hazards; and group 3 of 100 workers with continued exposure to occupational hazards. Both noise and total volatile organic compounds were significantly higher in workplaces for group 3. Significantly worse auditory thresholds at frequencies of 2, 3 and 4 kHz were found in group 3 compared to group 1 and group 2. The rate of prevalence of hearing loss was 23.5%, being the highest in group 3 (36.0%). The odds ratio of municipal workers of solid waste landfills associated with hearing loss was 3.39 (95% CI: 1.28–8.96) [41].

This study indicates that individuals with diabetes have 46% higher risk for hearing loss (RP_adjusted_ = 1:46: 95%CI: 1.32–1.61). In a study that evaluated predisposing factors for hearing loss [9] participants with diabetes *mellitus* showed significantly worse hearing results in 0.5, 1 and 6 kHz, which is confirmed in the population-based study of 16,040 individuals conducted in Korea [10], which verified that the risk of developing hearing loss in high frequency in people with diabetes is higher.

In this study, the clinical characteristics of the sample were adjusted only for age and sex for the presence of mild hearing loss. It was also performed logistic regression, and the odds ratio of diabetes for hearing loss was 1.42 (95% CI: 1.20–1.69) for mild impairment at high frequencies and 1.24 (95%CI: 1.05–1.45) for moderate to severe impairment at high frequencies. Only in these two cases it was observed statistical significance.

A recent meta-analysis has shown that the odds ratio of hearing impairment to the diabetic participants was 2.15 (95%CI:1.72–2.68) compared with non-diabetic participants and it is likely to be regardless of the effect of aging or the noisy environment to which one is exposed [42].

Another meta-analysis identified patients with type 2 diabetes with a significantly higher incidence of mild hearing loss compared to controls; thresholds in the average of pure tone audiometry were higher in diabetics for all frequencies, but were more clinically relevant in 6 and 8 kHz [43].

In a population survey of Bainbridge et al. [44] with data from 5140 participants aged 20–69 years, researchers used multivariate analysis adjusted for age, sex, race/ethnicity, education, income, exposure to recreational noise, exposure to occupational noise, military history, use of ototoxic drugs and smoking and the results showed than people with diabetes had greater odds ratios and statistically significant hearing loss in the worst and best ears in all degrees of hearing loss and frequencies.

The fact that the hearing impairment diagnosisis is self-reported, considering the perception of each person, can imply that mild hearing impairments are not identified as moderate or severe hearing impairmentby the subject, causing a possibility of measurement bias. Considering that most hearing impairments are progressive, a proportion of survey respondents may not have identified that has such a complaint, which explains the proportion of difference compared with other surveys using pure tone audiometry to estimate the prevalence of hearing impairment or any type of hearing impairment that could cause any disconfort.

Another fact that must also be taken into account when considering the prevalence of hearing impairment and its association with risk factors is whether it is congenital or acquired. In this study, the acquired hearing loss was more prevalent, making us think that the occupational exposure and the diseases acquired throughout life may influence the occurrence of this outcome, i.e., the more unhealthy the workplace is and the greater the diseases associated with hearing loss the person is exposed to, such as diabetes, the greater the prevalence of hearing impairment.

In the general population of PNS, the prevalence of diabetes was 6.2% (95% CI: 5.9–6.6), with higher prevalences among women, individuals over 60 years of age, non-smokers, non-alcoholics and people with lower educational levels. Among SHI cases, a similar profile was observed, except for the prevalence of diabetes in alcohol use, since there was no difference between use and non-use and in the educational variable, since higher prevalences were obtained in individuals with higher levels of schooling.

To diabetes there can be a difference between individuals with type 1 or type 2 diabetes, but considering that about 90.0% of cases are of type 2 diabetes [45], especially in the adult population, the results presented herein refer must predominantly to type 2 diabetes. Another factor known to be related to diabetes and consequently to hearing impairment is obesity, but this was not addressed in this study; it will be addressed in a future research.

The strengths of this study are the following: the size of the sample, because it has great statistical power and is representative of the Brazilian population; the response rate to the survey, which was very satisfactory (92%) and; with the weights, the results can be extrapolated to the entire Brazilian population, and perhaps the most important point is about the confounding variables, so that the factors associated with hearing loss were explained as clearly as possible, a factor observed in the multivariate analysis. This is the first Brazilian study we know that this aspect related to occupational exposure, such as noise, chemicals and solid waste, was considered in the multivariate analysis, along with tobacco and alcohol consumption, factors previously reported to be associated with hearing impairment.

On the other hand, that are several limitations of this study. The research refers to a cross-sectional study, and conclusions about statistical associations may not be causal, and reverse causality cannot be ruled out. In addition, as the exposures and outcome were based on self-reports, there may be a reporting bias, which may lead to inflated associations due to the variance of the common method.

## Conclusion

The results of the present study suggest that individuals with diabetes has higher prevalence of hearing impairment. The investigation of the association between non-communicable diseases, such as diabetes, and hearing impairment should be continued in order to provide a clearer understanding of the etiologic risk factors related to hearing impairment, resulting in the establishment of better actions for vulnerable populations. Thus, public health resources can be better applied, contributing to the development of public policies for the prevention of hearing loss. This study may also foster more targeted and planned research to investigate factors interconnected between the various short comings.

## Abbreviations

HI: Hearing Impairment
IBGE: Instituto Brasileiro de Geografia e Estatística
PNS: Pesquisa Nacional de Saúde
SHI: Self-reported Hearing Impairment
